# Less amputations for diabetic foot ulcer from 2008 to 2014, hospital management improved but substantial progress is still possible: A French nationwide study

**DOI:** 10.1371/journal.pone.0242524

**Published:** 2020-11-30

**Authors:** Coralie Amadou, Pierre Denis, Kristel Cosker, Anne Fagot-Campagna

**Affiliations:** 1 Paris-Saclay University, Corbeil-Essonnes, France; 2 Department of Diabetes and Endocrinology, Sud-Francilien Hospital, Corbeil-Essonnes, France; 3 Caisse Nationale d'Assurance Maladie (CNAM), French National Health Insurance, Paris, France; di Pompeo d'Illasi, Universita degli Studi di Roma La Sapienza Facolta di Medicina e Psicologia, ITALY

## Abstract

**Objective:**

To assess the improvement in the management of diabetes and its complications based on the evolution of hospitalisation rates for diabetic foot ulcer (DFU) and lower extremity amputation (LEA) in individuals with diabetes in France.

**Methods:**

Data were provided by the French national health insurance general scheme from 2008 to 2014. Hospitalisations for DFU and LEA were extracted from the SNIIRAM/SNDS French medical and administrative database.

**Results:**

In 2014, 22,347 hospitalisations for DFU and 8,342 hospitalisations for LEA in patients with diabetes were recorded. Between 2008 and 2014, the standardised rate of hospitalisation for DFU raised from 508 to 701/100,000 patients with diabetes. In the same period, the standardised rate of LEA decreased from 301 to 262/100,000 patients with diabetes. The level of amputation tended to become more distal. The proportion of men (69% versus 73%) and the frequency of revascularization procedures (39% versus 46%) increased. In 2013, the one-year mortality rate was 23% after hospitalisation for DFU and 26% after hospitalisation for LEA.

**Conclusions:**

For the first time in France, the incidence of a serious complication of diabetes, i.e. amputations, has decreased in relation with a marked improvement in hospital management.

## 1. Introduction

While the prevalence of diabetes is increasing in developed countries, the quality of management of diabetes is improving and the cost of management is increasing [1]. In France, the estimated prevalence of pharmacologically treated diabetes was 5.0% in 2016, within the mean range observed in Europe, with an adjusted (age and region) annual growth rate of + 0.9% and + 0.4%, respectively for men and women, between 2010 and 2017 [2]. In 2012, annual national health insurance expenditure related to diabetes was estimated to be €10 billion [3]. In parallel, marked improvement of the quality of care has been observed over recent years [4] as reflected by declining levels of HbA1c, blood pressure and dyslipidemia, although the prevalence of smoking has not decreased, and the prevalence of obesity is increasing among patients with diabetes in France. No reduction of the prevalence or incidence of complications of diabetes has been demonstrated in France, in contrast with other countries [5]. Diabetic foot complications are serious and costly [6]. Diabetic foot ulcer (DFU) most often results from the combination of two major complications of diabetes: diabetic neuropathy and arterial disease. It can be complicated by soft tissue and bone infection. Arterial disease represents the most severe prognosis in terms of amputation and survival [7]. Prevention remains the most effective weapon against DFU. Guidelines on screening and management of patients with diabetes at risk of foot ulcer have been published by various scientific societies, especially the International Working Group on the Diabetic Foot (IWGDF), since 2007 [8–10].

In France, patients with diabetes can be eligible for 100% health insurance coverage for healthcare related to diabetes. However, in 2007, the ENTRED study found that 23% of patients with type 1 diabetes and 17% of patients with type 2 diabetes had given up at least one health service over the past year because of its price, essentially dietetic, dental and podiatric care [11], which was not included in the 100% healthcare coverage. An outpatient podiatry allowance was therefore specifically set up in June 2008 to allow reimbursement of 4 podiatry sessions per year in the presence of grade 2 risk (sensory neuropathy associated with arterial disease and/or foot deformity) and 6 sessions per year in the presence of grade 3 risk of foot ulcer (history of foot ulcer and/or amputation). Over the same period, podiatrists have been provided with training and specific qualifications, and general practitioners have been provided with foot ulcer management guidelines and general management guidelines for diabetes and vascular risk.

The aim of this study was to analyze changes in the incidence of DFU, and the evolution of hospital and outpatient management of DFU between 2008 and 2014. This period corresponds indeed in a phase of improvement and standardisation in the medical management of DFU in France. This study was conducted on data from the national health insurance database (SNIIRAM/SNDS), a comprehensive and national database of healthcare reimbursement data from over 60 million people which is largely used for providing insight and help decision making [12].

## 2. Methods

### 2.1. Medical and administrative database

The SNIIRAM/SNDS is an anonymous individual database concerning all beneficiaries of the various national health insurance schemes in France [12]. Many published studies have been based on the SNIIRAM/SNDS, which is one of the largest medical administrative databases in the world and is largely used to guide public health policies in France [3,12–15].

It exhaustively records all reimbursed prescriptions and outpatient services and procedures, as well as their dates. Identification of medicinal products is based on the ATC code (Anatomical Therapeutic Classification), that of laboratory examinations is based on the national laboratory test coding table and that of procedures is based on the *Classification Commune des Actes Médicaux* (CCAM) [common classification of medical procedures]. The SNIIRAM does not contain any clinical data concerning the results related to prescriptions or examinations, but nevertheless includes information on the possible presence of long-term diseases (LTD), such as diabetes, which are eligible for 100% reimbursement of healthcare expenditure at the physician’s and patient’s request, following approval by a national health insurance physician. These LTDs are coded according to the international classification of diseases (ICD-10). A unique and anonymous identification number for each person also allows integration of the hospital discharge database (PMSI, *Programme de médicalisation des systèmes d’information*) into the SNIIRAM/SNDS database. The principal diagnoses and associated diagnoses recorded in the PMSI are coded according to ICD-10 and the procedures performed are coded according to CCAM.

### 2.2. Study population

#### 2.2.1. Identification of patients with diabetes and choice of study period

Each year, from 2008 to 2014, people to whom oral antidiabetic treatments or insulin were dispensed on at least 3 distinct dates (or at least 2 dates in the case of large pack sizes, corresponding to treatment for a period of 3 months) during year n or year n-1, or receiving LTD coverage for diabetes for year n were considered to have diabetes. The list of antidiabetic drugs corresponds to class A10 of the Anatomical Therapeutic Chemical (ATC) classification with the exclusion of benfluorex. The study period was chosen to start post IWGDF 2007 guidelines [8] because of restriction on access to SNDS data before 2006 which would therefore not have allowed a comparison before and after guidelines.

#### 2.2.2. Study of hospitalisations for diabetic foot ulcer and lower extremity amputation

The present study was limited to the population covered by the national health insurance general scheme, which is about 86% of the 65 million inhabitants in France, as it is the only scheme for which both vital status and LTD are comprehensively recorded during the study period.

#### 2.2.3. Study of 12-months post hospitalisation outcomes

From the initial study population (2.2.2.), we further excluded people insured by specific national health insurance general scheme contracts, i.e. mostly students and teachers, for whom vital status was not comprehensively recorded (10% of the overall population), thereby leaving 76% of the overall population. Therefore, analysis of outcomes before and after hospitalisation for amputation or foot ulcer were limited to people for whom the vital status was available and who had not been hospitalised during the previous 12 months in order to ensure an observation period of at least 24 months. Finally, analyses on podiatric and nursing care before and after amputation were limited to people still alive after 12 months and not readmitted to hospital for foot ulcer or amputation. These analyses were performed over 2 periods (patients hospitalised in 2010 and patients hospitalised in 2013, for whom data before and after hospitalisation were available) in order to study variations in the use of the new podiatry allowance.

#### 2.2.4. Nationwide use of the podiatry allowance

Finally, we assessed the level of nationwide use of the podiatry allowance with no restriction to a particular national health insurance scheme but based on SNIIRAM/SNDS data for all of France from 2010 to 2014.

### 2.3. Information collected

Hospitalisations for lower extremity amputations were identified by the procedures listed in S1 Table. Hospitalisations for foot ulcer were identified by the diagnoses listed in S2 Table regardless of the position of the diagnosis: principal, related or associated. Identified hospital revascularization procedures are listed in S3 Table. Podiatry allowances for patients at high risk of diabetic foot (grades 2 and 3) were identified by specific reimbursement of this allowance in the SNIIRAM database. People receiving a specific allowance granted by the State for low-income earners, or complementary universal health insurance cover [CMU-C]) or with a hospital diagnosis code including a precarity code (category Z59 of ICD-10 listed in S4 Table) were considered to present a social vulnerability marker. These indirect markers of social vulnerability were specifically studied in people under the age of 60 years, as, beyond this age, these patients may be eligible for other social security benefits, not recorded in the SNIIRAM/SNDS. Nursing care allowances (coefficient equal to 4 including dressings, but without being able to specifically isolate these procedures from infusions and injections) were identified from among outpatient procedures. Comorbidities were identified by using algorithms developed by the *Caisse nationale d’assurance maladie des travailleurs salariés* (salaried workers national health insurance fund) from the SNIIRAM/SNDS data, LTD diagnoses, hospital stays and, in some cases, drugs or specific medical or therapeutic procedures [16].

### 2.4. Statistical analysis

Descriptive analyses focus on the exhaustiveness of national health insurance general scheme data and consequently do not include calculation of confidence intervals. The evolution of the number of patients with diabetes hospitalised for lower extremity amputation or foot ulcer were observed between 2008 and 2014. Annual rates were standardised according to the age and sex structure of the population with diabetes covered by the national health insurance general scheme in 2008. Extrapolation to the general population of France as a whole was performed for 2014 from the Institut National de la Statistique et des Etudes Economiques (INSEE) population 2014 [17] adjusted to sex and age population structure.

Data extraction and statistical analyses were performed with SAS Enterprise Guide version 4.3. software.

Analyses of the SNIIRAM/SNDS databases have been approved by the French personal data protection agency (*Commission Nationale Informatique et Libertés*).

## 3. Results

### 3.1. Evolution of incidence and characteristics of lower extremity amputations

**Fig 1:**
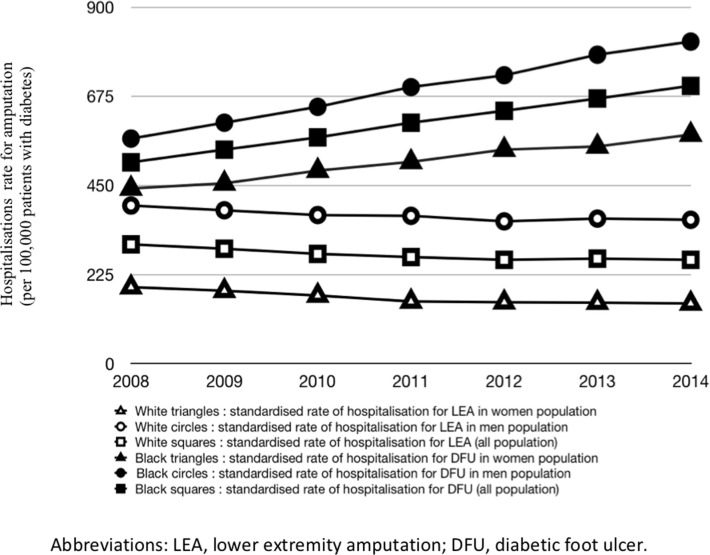
Variations from 2008 to 2014 of the rates of patients with diabetes hospitalised for foot ulcer or lower extremity amputation (per 100,000 patients with diabetes), standardised to the age and sex structure of the 2008 population with diabetes. Data from the SNIIRAM/SNDS database.

Hospitalisation rates for lower extremity amputation or foot ulcer and their time-course from 2008 to 2014 are given in Table 1 and Fig 1.

**Table 1:** Numbers of hospital stays and patients with diabetes hospitalised for foot ulcer and lower extremity amputation, crude rates and standardised rates according to the age and sex structure of the population with diabetes in 2008 (for 100,000 patients with diabetes) from 2008 to 2014. Data from the SNIIRAM/SNDS database.

Year		*2008*	*2009*	*2010*	*2011*	*2012*	*2013*	*2014*
Patients with diabetes		2,395,525	2,529,095	2,685,423	2,787,257	2,889,900	2,978,976	3,063,372
**DFU**	Number of stays	22,429	24,861	27,189	29,109	31,201	33,661	36,58
	Number of people	12,174	13,723	15,458	17,219	18,917	20,586	22,347
	Crude rate	508	543	576	618	655	691	730
	Standardised rate for women	442	455	487	509	540	548	578
	Standardised rate for men	568	608	648	698	728	780	813
	Standardised rate for all	508	540	571	608	638	669	701
**LEA**	Number of stays	9,005	9,311	9,408	9,527	9,672	10,217	10,458
	Number of people	7,205	7,385	7,52	7,635	7,742	8,130	8,342
	Crude rates	301	292	280	274	268	273	272
	Standardised rate for women	193	184	172	157	155	154	152
	Standardised rate for men	399	387	375	373	359	366	363
	Standardised rate for all	301	290	277	269	262	265	262

Abbreviations: LEA, lower extremity amputation; DFU, diabetic foot ulcer.

Between 2008 and 2014, the number of patients on treatment for diabetes covered by the national health insurance general scheme increased by about 670,000 people. In 2014, more than 3,000,000 people were covered by this scheme and treated for diabetes. The number of these patients with diabetes hospitalised for DFU in 2014 was 22,347, almost twice that observed in 2008. The number of hospitalisations for lower extremity amputation was 8,342 in 2014, i.e. 1,137 more patients than in 2008. Extrapolated to the overall population of France, it represented 3,538,106 patients on treatment for diabetes, including 26,094 patients hospitalised for foot ulcer in 2014 and 9,778 patients hospitalised for lower extremity amputation. The standardised incidence rate (age and sex) of patients with diabetes hospitalised for foot ulcer increased from 508 to 701 per 100,000 (+193 points). In contrast, the standardised incidence rate (age and sex) of lower extremity amputation (LEA) over the same period decreased from 301 to 262 per 100,000 patients with diabetes (-39 points).

In 2014, amputations involved the thigh in 15% of cases (-2 points since 2008), the leg in 18% of cases (-1 point), and the foot in 18% of cases (+1 point). The proportion of toe amputations was 49% in 2014 (+ 2 points).

### 3.2. Characteristics of hospitalised patients

The mean age of patients with diabetes hospitalised for foot ulcer increased slightly from 70 to 72 years between 2008 and 2014 while it remained stable at 71 years in patients hospitalised for lower extremity amputation. The proportion of men among patients hospitalised for foot ulcer and amputation increased respectively to 61% (+ 3 points between 2008 and 2014) and 73% (+ 4 points). Respectively, 97% and 83% of patients hospitalised for foot ulcer or amputation received 100% health insurance coverage for either LTD diabetes alone or for any chronic disease, while all were eligible.

A revascularization procedure had been performed during the year in 20% of patients hospitalised for foot ulcer (+4 points) and in 46% of patients hospitalised for amputation (+7 points since 2008). Patients with diabetes hospitalised for foot ulcer or amputation in 2014 frequently presented another serious complication of diabetes. Respectively (foot ulcer and amputation): 79% and 89% had marker of cardiovascular and neurovascular diseases and 6% and 13% were treated for end-stage renal disease. Furthermore, markers of chronic respiratory disease were present in 23% and 19% of patients, cancer in 17% and 16% of patients, neurological or degenerative disease in 13% and 12% of patients, liver or pancreatic disease in 11% and 9% of patients, and psychiatric illness in 8% and 6% of patients.

### 3.3. Patients with diabetes under the age of 60 years (social characteristics)

Patients under the age of 60 years represented 17% of patients with an amputation. Among patients hospitalised for amputation in 2014, 41% presented a marker of social vulnerability (+ 11 points compared to 2010—variable unavailable in 2008 and 2009). Among those hospitalised for foot ulcer in 2014, 39% presented a marker of social vulnerability (+ 8 points).

### 3.4. Outcome of patients with diabetes after hospitalisation for foot ulcer or lower extremity amputation (Table 2)

**Table 2:** Outcomes 12 months after hospitalisation for lower extremity amputation or foot ulcer in 2013 among patients with diabetes by age group. Data from the SNIIRAM/SNDS database.

		< 55 years	56–64 years	65–74 years	≥ 75 years	Total
After hospitalisation for DFU	Number of patients	1,527	2,89	3,772	7,016	15,205
	Readmission for DFU (% of age group)	566 (37)	1166 (40)	1444 (38)	2,246 (32)	5,422 (36)
	Readmission for LEA (% of age group)	338 (22)	751 (26)	904 (24)	1,191 (17)	3,184 (21)
	Readmission for all other causes *(% of age group)	816 (53)	1640 (57)	2237 (59)	4,050 (58)	8,743 (58)
	Death (% of age group)	79 (5)	306 (11)	717 (19)	2,417	3,519 (23)
After hospitalisation for LEA	Number of patients	441	1,064	1,329	2,009	4,843
	Readmission for DFU (% of age group)	236 (54)	518 (49)	612 (46)	722 (36)	2,088 (43)
	Readmission for LEA (% of age group)	95 (22)	293 (28)	377 (28)	544 (27)	1,309 (27)
	Readmission for all other causes* (% of age group)	239 (54)	601 (56)	826 (62)	1147 (57)	2,813 (58)
	Death (% of age group)	24 (5)	148 (14)	266 (20)	810 (40)	1,248 (26)

Table reading: five percent of patients of the « under 55 years » group hospitalised for diabetic foot ulcer died in the 12 months following hospitalisation.

One patient can be recorded several times as he/she can be hospitalised several times during the 12 months.

Abbreviations: LEA, lower extremity amputation; DFU, diabetic foot ulcer.

* Except amputation or foot ulcer

In the population hospitalised for foot ulcer in 2013 (without hospitalisation for amputation or foot ulcer during the previous 12 months), 23% died during the following 12 months, at a mean age of 79 years. Twelve-month mortality rates (22% for the 2010 cohort versus 23% for the 2013 cohort) and amputation rates (21% in both cohorts), for patients hospitalised for foot ulcer in 2013, were almost identical to those of patients hospitalised for foot ulcer in 2010, but the readmission rate for foot ulcer was slightly higher in 2013 (34% in 2010 versus 36% in 2013).

In the population hospitalised for lower extremity amputation in 2013 (without hospitalisation for amputation or foot ulcer during the previous 12 months), 26% died during the following 12 months, at a mean age of 77 years. After an amputation, one half (56%) of patients were readmitted to hospital, at least once for foot ulcer or amputation, (with or without death) during the 12 months following the first hospitalisation: 27% of patients were readmitted for amputation and 43% for foot ulcer. The all-cause readmission rate, excluding amputation or foot ulcer, was 58% (one patient could be recorded several times as he/she could be hospitalised several times during the 12 months).

Twelve-month mortality rates (27% for the 2010 cohort versus 26% for the 2013 cohort) and re-amputation rates (27% in both cohorts) for patients hospitalised for lower extremity amputation in 2013 were almost identical to those of patients hospitalised for lower extremity amputation in 2010, but the readmission rate for foot ulcer was higher in 2013 (35% in 2010 versus 43% in 2013).

### 3.5. Use of the podiatry and nursing care allowances before and after hospitalisation for foot ulcer or lower extremity amputation (Table 3)

**Table 3:** Use of podiatry and nursing care allowances before and after hospitalisation for DFU or lower extremity amputation in 2010 and 2013. Data from the SNIIRAM/SNDS database.

			Hospitalisation for LEA in 2010	Hospitalisation for DFU in 2010	Hospitalisation for LEA in 2013	Hospitalisation for DFU in 2013
Use of the outpatient podiatry allowance	During the 12 months preceding hospitalisation	% patients using the allowance	12	13	20	20
		Mean number of sessions for people using the allowance	2.6	2.7	2.7	2.9
	During the 12 months following hospitalisation*	% patients using the allowance	19	19	28	26
		Mean number of sessions for people using the allowance	2.9	3.1	2.9	3.1
Use of the outpatient nursing care allowance	During the 12 months preceding hospitalisation	% patients using the allowance	42	45	49	50
		Mean number of sessions for people using the allowance	63	65	70	93
	During the 12 months following hospitalisation*	% patients using the allowance	60	54	61	55
		Mean number of sessions for people using the allowance	96	104	93	102

* without hospitalisation for foot ulcer or amputation during the previous 12 months)

** among surviving patients not readmitted to hospital for amputation or foot ulcer

Abbreviations: DFU, diabetic foot ulcer; LEA, lower extremity amputation.

In the population hospitalised for foot ulcer in 2013 (without hospitalisation for foot ulcer or amputation during the previous 12 months), the outpatient podiatry allowance was used by 20% of patients before hospitalisation, with an average of 2.9 sessions per year, and by 26% of patients after hospitalisation (after exclusion of deaths and readmissions for amputation or foot ulcer), with an average of 3.1 sessions per year. Use of the podiatry allowance also increased in 2013 compared to 2010, when it was used by 13% of patients before hospitalisation with an average of 2.7 sessions per patient, and by 19% of patients after hospitalisation with an average 3.1 sessions per patient.

In the population hospitalised for lower extremity amputation in 2013 (without hospitalisation for foot ulcer or amputation during the previous 12 months), the outpatient podiatry allowance was used by 20% of patients during the 12 months prior to hospitalisation, with an average of 2.7 sessions per year, and by 28% of patients (after exclusion of deaths and readmissions for amputation or foot ulcer) after hospitalisation, with an average of 2.9 sessions per year. Use of the podiatry allowance increased in 2013 compared to 2010, when it was used by 12% of patients before hospitalisation with an average of 2.6 sessions per patient, and 19% of patients after hospitalisation, with an average of 2.9 sessions per patient.

In the population hospitalised for foot ulcer in 2013, outpatient nursing care, which can also be related to foot care, was used by 50% of patients before hospitalisation and 55% of patients after hospitalisation, representing a slight increase compared to the figures observed in 2010 (45% before hospitalisation and 54% after hospitalisation).

In the population hospitalised for amputation in 2013, outpatient nursing care was used by 49% of patients before hospitalisation and 61% of patients after hospitalisation, representing a slight increase compared to the figures observed in 2010 (42% before hospitalisation and 60% after hospitalisation).

### 3.6. Nationwide use of the podiatry allowance

In 2014, 9,500 podiatrists (versus 6,980 in 2010) billed at least one procedure covered by the podiatry allowance for at least one patient with diabetes. Collectively, these podiatrists had cared for a total of 250,000 patients with diabetes in 2014 (versus 96,700 in 2010), i.e. an average of 26 (versus 14) patients per podiatrist per year with an average of 2.7 (versus 2.5) sessions per patient and per year, versus the recommended 4 or 6 sessions. Patients cared for in the context of the podiatry allowance were predominantly (54%) women with a mean age of 65 years.

## 4. Discussion

### 4.1. Hospitalisations for DFU and amputations

This study documents for the first time in France a declining incidence of a serious complication of diabetes, lower extremity amputation, in parallel with increasing rates of hospitalisation for foot ulcer. Although hospital management markedly improved between 2008 and 2014, outpatient management remains insufficient despite the creation of a podiatry allowance in 2008, which allows 100% reimbursement of 4 to 6 sessions per year for patients with diabetes at high risk of foot ulcer.

Our results are consistent with those observed in several countries such as the USA (4), The Netherlands [18], Scotland [19], Finland [20], Australia [21,22], Denmark [23] and Belgium [24] and confirm the decrease in LEA among people with diabetes, as it was also reported more recently by Harding et al. [25]. Even if the trend is decreasing, not all results are comparable because of the differences in periods and population structure. Overall, the decrease in the incidence rate of amputations in patients with diabetes in France is less rapid than in the studies mentioned above. In comparison with Belgium [24], which is a country very comparable to France, and for which the study period is almost the same as the present study, the incidence rate of amputation is about 2.5 times higher in France, and the decrease in the incidence rate of amputations two times longer in France. Despite the fact that our study only started in 2008, we can still mention results published previously by Fosse et al. [6] showing a crude LEA rate of 378/100 000 patients with diabetes in France in 2003 vs 301 and 272/100 000 respectively in 2008 and 2014.

In France, a marked improvement in the general care of patients with diabetes has been confirmed by the national ENTRED studies [4]. These studies demonstrated an improvement of cardiovascular risk factor control and increased life expectancy between 2001 and 2007, prior to the period studied here. However, it is difficult to estimate the lag-time between better risk control and a visible impact on the development of serious complications of diabetes.

This declining incidence of lower extremity amputations related to diabetes has been observed following the implementation of several actions in France. The present study also shows an increasing proportion of more distal and therefore less traumatic amputations, as well as an increasing proportion of revascularization procedures allowing to avoid some amputations or better healing after amputation. In parallel, the rate of hospitalisations for foot ulcer is increasing.

### 4.2. Prevention remains insufficient

The setting-up of a podiatry allowance allowing 100% coverage of 4 to 6 podiatry sessions per year for high-risk subjects is specific to the French national health insurance and completes the 100% reimbursement of all medical expenditure related to diabetes or other chronic diseases. The use of outpatient podiatric care remains globally insufficient, and the number of sessions performed remains inadequate. The inadequate rates of outpatient podiatric care could be explained by the progressive take-up rate of the podiatry allowance, but also by the patient payment, prior to reimbursement by national health insurance, which should disappear with improvement in the electronic system, and possibly also poorer adhesion with recommended care by some patients. The podiatry allowance is also predominantly used by women, despite the fact that men are at greater risk. Several additional measures are currently being adopted in France to reinforce the outpatient management of DFU. French national health insurance has developed tools designed for general practitioners, including an electronic application on chronic wounds designed to more widely diffuse good clinical practice guidelines to healthcare professionals (« e-mémo plaies chroniques », available on iOS and Android). A program designed to provide support for home care (PRADO) after hospitalisation for chronic wounds, pressure ulcers, venous ulcers or DFU, is also currently under evaluation [26].

Finally, the development of centers specialized in highly specific management targeting a small number of patients at very high risk of mortality constitutes a major challenge for the healthcare system. Also, the improvement and research of prevention techniques continues. For example, there are encouraging results concerning nerve decompression in patients already suffering from diabetic neuropathy. But more data and randomized studies are still needed [27].

### 4.3. Prognosis and risk factors

Several other aspects of this study need to be highlighted. First of all, the reduction of the amputation rate appears to be less marked over recent years and the number of patients with diabetes requiring amputation is continuing to increase as a result of demographic growth and other factors.

Secondly, over this seven-year period, amputations tended to become concentrated among men, particularly among younger men with a marker of social vulnerability. The high amputation rate in men associated with unfavorable socioeconomic conditions has been investigated in several studies [20,28,29]. This association raises the important issue of access to prevention and improvement of the management of the most vulnerable patients, even within the specific framework of 100% reimbursement of medical expenses.

Lastly, our study reveals a one-year mortality of 23% and 26% after hospitalisation for foot ulcer or amputation, respectively. In 2019, Amadou et al. [7] described the survival of a cohort of 347 patients with DFU treated in a French reference center between 2009 and 2010. The one-year mortality was 10% with no significant difference between in and outpatients. Despite differences in the methodology of survival probability calculation, this major difference in survival between national results and a reference center should alert us of the differences in patient’s prognosis within the same country.

### 4.4. Database limits

The main strength of this study concerns the use of the SNIIRAM/SNDS database, which allows comprehensive follow-up of a very large population of patients with diabetes among general scheme beneficiaries, i.e. 86% of the population living in France. Although the method used in this study is not subject to the biases of declarative surveys, it nevertheless presents a number of classical limitations related to data derived from medical and administrative databases. As diabetes was defined by reimbursements for antidiabetic drugs or long-term disease status for diabetes, patients with diabetes treated by diet alone and those hospitalised in certain institutions for dependent elderly subjects in which drugs can be dispensed without individual billing, were not detected. Furthermore, detection of hospitalisations for amputation or foot ulcer was based on hospital coding of diagnoses and the procedures performed: the link between these hospitalisations and the presence of diabetes is usually reported, but some of these hospitalisations could have been related to trauma (instead of diabetic foot ulcer). Nevertheless, a previous study demonstrated an excess of amputations for traumatic causes in patients with diabetes compared to nondiabetic patients [5], highlighting the importance of analyzing all amputations, regardless of their cause, in the context of diabetes.

## 5. Conclusion

For the first time in France, as already shown in other countries, this study demonstrates a reduction of the incidence of lower extremity amputation, which constitutes a serious complication of diabetes, but substantial progress is still possible in comparison to other countries. In parallel, hospitalisation rates for foot ulcer have markedly increased. These changing rates suggest a marked improvement of management of diabetic foot complications, but still at an excessively advanced stage. In a country in which the management of diabetes and podiatric care can be performed with no financial cost to the patient, outpatient management nevertheless remains insufficient. The essential challenge consists of rapidly delivering good quality specialized care, throughout the country, to a small, very frail, and often financially precarious population, which remains socially poorly accessible. In addition to social inequality mentioned above, more consistent standardisation of medical practices must be considered in order to provide an equality of opportunity for patients with diabetes regardless of their care center.

## Supporting information

S1 Table Procedures used to identify hospitalisations for lower extremity amputations in the SNIIRAM database. (DOCX)

S2 Table Diagnoses in the SNIIRAM database used to identify hospitalisations for foot ulcer (at least one diagnosis during the hospital stay, regardless of its position: Principal, related or associated). (DOCX)

S3 Table List of hospital revascularization procedures identified in the SNIIRAM database. (DOCX)

S4 Table ICD-10 codes of the Z59 category: Problems related to housing and economic circumstances. (DOCX)

## Abbreviations

DFU: diabetic foot ulcer
LEA: lower extremity amputation
SNDS: système national des données de santé (National Heath data system)
SNIIRAM: Système national d'information inter-régimes de l'Assurance maladie (National inter-scheme health insurance information system)
