# Supplementary material for: Less amputations for diabetic foot ulcer from 2008 to 2014, hospital management improved but substantial progress is still possible: A French nationwide study
Source: PLoS One. 2020 Nov 30;15(11):e0242524. doi: 10.1371/journal.pone.0242524 (PMC7703996; doi:10.1371/journal.pone.0242524)
Supplement: S1 Table — (DOCX) [file pone.0242524.s001.docx]

**S1-Table.** Procedures used to identify hospitalisations for lower extremity amputations in the SNIIRAM database

| **CCAM code** | **Description** |
| --- | --- |
| NZFA004 | Amputation or disarticulation of several toes |
| NZFA010 | Amputation or disarticulation of one toe |
| NZFA005 | Amputation or disarticulation at the midfoot or forefoot, without stabilization of the hindfoot |
| NZFA009 | Amputation or disarticulation at the ankle or hindfoot |
| NZFA013 | Amputation or disarticulation of the midfoot or forefoot, with stabilization of the hindfoot |
| NZFA002 | Transtibial amputation |
| NZFA003 | Disarticulation of the knee |
| NZFA001 | Disarticulation of the hip |
| NZFA006 | Lower extremity disarticulation or amputation through the hip bone, sacro-iliac joint or sacrum |
| NZFA007 | Transfemoral amputation |
| NZFA008 | Interilioabdominal disarticulation or amputation |
