# Supplementary material for: Less amputations for diabetic foot ulcer from 2008 to 2014, hospital management improved but substantial progress is still possible: A French nationwide study
Source: PLoS One. 2020 Nov 30;15(11):e0242524. doi: 10.1371/journal.pone.0242524 (PMC7703996; doi:10.1371/journal.pone.0242524)
Supplement: S2 Table — (DOCX) [file pone.0242524.s002.docx]

**S2 Table**. Diagnoses in the SNIIRAM database used to identify hospitalisations for foot ulcer (at least one diagnosis during the hospital stay, regardless of its position: principal, related or associated)

| **ICD-10 code** | **Description** |
| --- | --- |
| L97 | Ulcer of lower limb, not elsewhere classified |
| S90 | Superficial injury of ankle and foot |
| S91 | Open wound of ankle and foot |
| M86.07 | Acute haematogenous osteomyelitis - Ankle and foot |
| M86.17 | Other acute osteomyelitis - Ankle and foot |
| M86.27 | Subacute osteomyelitis - Ankle and foot |
| M86.37 | Chronic multifocal osteomyelitis - Ankle and foot |
| M86.47 | Chronic osteomyelitis with draining sinus - Ankle and foot |
| M86.57 | Other chronic haematogenous osteomyelitis - Ankle and foot |
| M86.67 | Other chronic osteomyelitis - Ankle and foot |
| M86.87 | Other osteomyelitis - Ankle and foot |
| M86.97 | Osteomyelitis, unspecified - Ankle and foot |
