# Supplementary material for: Less amputations for diabetic foot ulcer from 2008 to 2014, hospital management improved but substantial progress is still possible: A French nationwide study
Source: PLoS One. 2020 Nov 30;15(11):e0242524. doi: 10.1371/journal.pone.0242524 (PMC7703996; doi:10.1371/journal.pone.0242524)
Supplement: S3 Table — (DOCX) [file pone.0242524.s003.docx]

**S3-Table.** List of hospital revascularization procedures identified in the SNIIRAM database

| **CCAM code** | **Description** |
| --- | --- |
| DGAF005 | Percutaneous transluminal angioplasty of the abdominal aorta with stent placement |
| DGAF008 | Percutaneous transluminal angioplasty of the abdominal aorta without stent placement |
| DGCA003 | Direct retroperitoneal bypass graft between the descending thoracic aorta and femoral arteries |
| DGCA004 | Bifurcated aortobifemoral bypass graft via laparotomy with infrarenal clamping |
| DGCA007 | Infrarenal aortoaortic bypass graft via laparotomy with infrarenal clamping |
| DGCA009 | Unilateral aortofemoral bypass graft via laparotomy with infrarenal clamping |
| DGCA010 | Bifurcated aortobifemoral bypass graft via laparotomy with suprarenal clamping |
| DGCA012 | Infrarenal aortoaortic bypass graft via laparotomy with suprarenal clamping |
| DGCA019 | Unilateral aortofemoral bypass graft via laparotomy with suprarenal clamping |
| DGCA020 | Bifurcated aorto-ilio-femoral bypass graft via laparotomy with suprarenal clamping |
| DGCA022 | Bifurcated aortobiiliac bypass graft via laparotomy with infrarenal clamping |
| DGCA026 | Bifurcated aortobiiliac bypass graft via laparotomy with suprarenal clamping |
| DGCA029 | Bifurcated aorto-ilio-femoral bypass graft via laparotomy with infrarenal clamping |
| DGCA030 | Repeated [redo] bifurcated aortobifemoral bypass graft via laparotomy without stent removal |
| DGFA001 | Abdominal aorta thromboendarterectomy via laparotomy |
| DGFA003 | Aortobiiliac thromboendarterectomy via laparotomy |
| DGFA004 | Abdominal aorta, common iliac artery and/or external iliac artery thrombectomy via a bilateral inguinofemoral approach |
| DGFA005 | Thrombectomy of a bypass graft or thromboendarterectomy of the abdominal aorta or its branches via laparotomy |
| DGFA007 | Unilateral aorto-ilio-femoral thromboendarterectomy via laparotomy |
| DGFA008 | Bilateral aorto-ilio-femoral thromboendarterectomy via laparotomy |
| DGFA009 | Unilateral aorto-iliac thromboendarterectomy via laparotomy |
| DGFA010 | Abdominal aorta, common iliac artery and/or external iliac artery thrombectomy via laparotomy |
| DGFA012 | Thrombectomy of a bypass graft or thromboendarterectomy of the abdominal aorta or its branches via an inguinofemoral approach |
| DGFA015 | Resection-anastomosis of the abdominal aorta or common iliac artery via laparotomy |
| DGLF001 | Percutaneous transluminal bifurcated aortobiiliac drug-eluting stent placement |
| DGLF002 | Percutaneous transluminal aortouniiliac drug-eluting stent placement |
| DGLF005 | Percutaneous transluminal infrarenal abdominal aorta linear drug-eluting stent placement |
| DGPA001 | Repair of aorto-ilio-femoral aneurysm with bifurcated aorto-ilio-femoral tube prosthesis via laparotomy with suprarenal clamping |
| DGPA005 | Repair of non-ruptured infrarenal aortic aneurysm with infrarenal aortoaortic tube prosthesis via laparotomy with infrarenal clamping |
| DGPA008 | Repair of non-ruptured infrarenal aortic or aortobiiliac aneurysm with aortobiiliac tube prosthesis via laparotomy with suprarenal clamping |
| DGPA010 | Repair of non-ruptured infrarenal aortic or aortobiiliac aneurysm with aortobifemoral tube prosthesis via laparotomy with infrarenal clamping |
| DGPA012 | Repair of non-ruptured infrarenal aortic or aortobiiliac aneurysm with aortobiiliac tube prosthesis via laparotomy with infrarenal clamping |
| DGPA013 | Repair of non-ruptured infrarenal aortic or aortobiiliac aneurysm with aortobifemoral tube prosthesis via laparotomy with suprarenal clamping |
| DGPA016 | Repair of aorto-ilio-femoral aneurysm with bifurcated aorto-ilio-femoral tube prosthesis via laparotomy with infrarenal clamping |
| DGPA017 | Repair of non-ruptured infrarenal aortic aneurysm with infrarenal aortoaortic tube prosthesis via laparotomy with suprarenal clamping |
| DGPF001 | Percutaneous transluminal disobstruction of the aortic bifurcation |
| DGPF002 | Bilateral percutaneous transluminal revascularization of the aortic bifurcation with stent placement |
| EDAF002 | Percutaneous transluminal dilatation of the common iliac artery and/or external iliac artery without stent placement |
| EDAF003 | Percutaneous transluminal dilatation of the common iliac artery and/or external iliac artery with stent placement |
| EDAF004 | Percutaneous transluminal dilatation of the internal iliac artery without stent placement |
| EDAF006 | Percutaneous transluminal dilatation of the internal iliac artery with stent placement |
| EDCA003 | Direct crossover ilio-iliac, iliofemoral or femorofemoral artery bypass graft |
| EDFA003 | Iliofemoral thromboendarterectomy via laparotomy |
| EDFA006 | Iliac thromboendarterectomy via laparotomy |
| EDFA007 | Iliofemoral thromboendarterectomy via an inguinofemoral approach |
| EDLF004 | Percutaneous transluminal drug-eluting stent placement in the common iliac artery and/or external iliac artery with embolization of the internal iliac artery |
| EDLF005 | Percutaneous transluminal drug-eluting stent placement in the common iliac artery and/or external iliac artery |
| EDLF007 | Percutaneous transluminal drug-eluting stent placement in the internal iliac artery or a non-gastrointestinal branch of the abdominal aorta |
| EDPA001 | Repair of iliac aneurysm with unilateral ilio-iliac or iliofemoral tube prosthesis via laparotomy |
| EDPA005 | Repair of iliac aneurysm with unilateral aorto-iliac or aortofemoral tube prosthesis via laparotomy |
| EDPF001 | Percutaneous transluminal revascularization of the internal iliac artery with stent placement |
| EDPF006 | Percutaneous transluminal revascularization of the common iliac artery and/or external iliac artery with drug-eluting stent placement |
| EDPF007 | Percutaneous transluminal revascularization of the internal iliac artery without stent placement |
| EDPF008 | Percutaneous transluminal revascularization of the common iliac artery and/or external iliac artery without stent placement |
| EDPF009 | Percutaneous transluminal revascularization of the common iliac artery and/or the external iliac artery with stent placement |
| EEAF001 | Percutaneous transluminal dilatation of several lower extremity arteries without stent placement |
| EEAF002 | Percutaneous transluminal dilatation of a lower extremity artery with intraluminal dilatation of the ipsilateral common iliac artery and/or external iliac artery with stent placement |
| EEAF003 | Percutaneous transluminal dilatation of a lower extremity artery without stent placement |
| EEAF004 | Percutaneous transluminal dilatation of a lower extremity artery with stent placement |
| EEAF005 | Percutaneous transluminal dilatation of a lower extremity artery with intraluminal dilatation of the ipsilateral common iliac artery and/or external iliac artery common without stent placement |
| EEAF006 | Percutaneous transluminal dilatation of several lower extremity arteries with stent placement |
| EECA001 | Direct above-the-knee femoropopliteal artery bypass graft |
| EECA002 | Direct ipsilateral femorofemoral artery bypass graft |
| EECA003 | Direct below-the-knee femoropopliteal artery bypass graft |
| EECA005 | Direct foot artery bypass graft |
| EECA006 | Direct subclaviofemoral or axillobifemoral artery bypass graft |
| EECA007 | Direct subclaviofemoral or axillofemoral artery bypass graft |
| EECA008 | Direct femorotibial or femoroperoneal artery bypass graft without venous cuff |
| EECA010 | Direct femorotibial or femoroperoneal artery bypass graft with venous cuff |
| EDCA004 | Direct iliofemoral artery bypass graft for anastomotic complication of a prosthesis of the femoral bifurcation |
| EECA012 | Direct multiple sequential or bifurcated lower extremity artery bypass grafts |
| EDCA005 | Direct ipsilateral iliofemoral artery bypass graft |
| EEFA001 | Direct thromboendarterectomy of the femoral artery and/or its branches |
| EEFA002 | Transpopliteal thrombectomy of a lower extremity artery |
| EEFA003 | Direct thromboendarterectomy of the popliteal artery |
| EEFA004 | Thrombectomy of a lower extremity artery via an inguinofemoral approach |
| EEFA006 | Direct resection-anastomosis of a lower extremity artery |
| EEGA001 | Direct resection of a lower extremity artery prosthesis without revascularization |
| EEGA002 | Direct resection of a lower extremity artery prosthesis with revascularization |
| EEJF001 | Percutaneous transluminal thromboaspiration of a lower extremity artery or artery bypass graft |
| EEKA001 | Direct replacement or repair of a lower extremity artery |
| EELF002 | Percutaneous transluminal drug-eluting stent placement in a lower extremity artery |
| EEAA002 | Direct enlargement angioplasty of a lower extremity artery bypass graft |
| EENF001 | Percutaneous transluminal superselective in situ fibrinolysis of a lower extremity artery or artery bypass graft |
| EENF002 | Percutaneous transluminal selective or highly selective in situ fibrinolysis of a lower extremity artery or artery bypass graft |
| EEPF001 | Percutaneous transluminal revascularization of a lower extremity artery with stent placement |
| EEPF002 | Percutaneous transluminal revascularization of a lower extremity artery without stent placement |
| ENAF001 | Percutaneous transluminal dilatation of a non-anatomical lower extremity artery bypass graft with stent placement |
| ENAF002 | Percutaneous transluminal dilatation of a non-anatomical lower extremity artery bypass graft without stent placement |
| ENFA001 | Direct thrombectomy of a lower extremity artery bypass graft with anastomosis repair without extension of the bypass graft |
| ENFA004 | Direct thrombectomy of a lower extremity artery bypass graft with anastomosis repair and extension of the bypass graft |
| ENFA005 | Direct change of lower extremity artery bypass graft with arterial thrombectomy |
| ENFA006 | Direct thrombectomy of a lower extremity artery bypass graft without anastomosis repair |
| ENFF001 | Percutaneous transluminal thrombectomy or mechanical embolectomy of a non-anatomical lower extremity artery bypass graft |
| ENNF001 | Percutaneous transluminal in situ fibrinolysis of a non-anatomical lower extremity artery bypass graft |
| DGGA002 | Direct ablation of an abdominal aorta prosthesis with axillobifemoral bypass graft |
| DGGA003 | Ablation of an abdominal aorta prosthesis with aortobiiliac or aortobifemoral bypass graft via laparotomy |
| DGKA004 | Abdominal aorta or common iliac artery replacement via laparotomy |
| DGPA018 | Repair of ruptured infrarenal or aortobiiliac aortic aneurysm with tube prosthesis via laparotomy |
| DHCA004 | Ilio-iliac or iliocaval vein bypass graft via laparotomy |
| DHFA003 | Iliac vein and/or inferior vena cava thrombectomy via laparotomy |
| EDEA001 | Reimplantation or bypass graft of the internal iliac artery or one of its branches via laparotomy |
