# Supplementary material for: Less amputations for diabetic foot ulcer from 2008 to 2014, hospital management improved but substantial progress is still possible: A French nationwide study
Source: PLoS One. 2020 Nov 30;15(11):e0242524. doi: 10.1371/journal.pone.0242524 (PMC7703996; doi:10.1371/journal.pone.0242524)
Supplement: S4 Table — (DOCX) [file pone.0242524.s004.docx]

**S4-Table.** ICD-10 codes of the Z59 category: Problems related to housing and economic circumstances

| **ICD-10 code** | **Descriptions** |  |
| --- | --- | --- |
| Z59.0 | Homelessness |  |
| Z59.1 | Inadequate housing |  |
| Z59.2 | Discord with neighbours, lodgers and landlord |  |
| Z59.3 | Problems related to living in residential institution |  |
| Z59.4 | Lack of adequate food |  |
| Z59.5 | Extreme poverty |  |
| Z59.6 | Low income |  |
| Z59.7 | Insufficient social insurance and welfare support |  |
| Z59.8 | Other problems related to housing and economic circumstances |  |
| Z59.9 | Problem related to housing and economic circumstances, unspecified |  |
